# Assessing microbial diversity in open-pit mining: Metabarcoding analysis of soil and pit microbiota across operational and restoration stages

**DOI:** 10.1371/journal.pone.0320923

**Published:** 2025-04-07

**Authors:** Priscila Heredia Reto, Rosita Castillo Rogel, Gabriela Palomino Lucano, Jean Louis Falen, Ricardo David Avellan Laguno, Karina Zapata Vidaurre, Marisol Saavedra Febre, Gabriel Reyes Calle, Juan Zingg Rosell, Jimmy Lopez Perez, José Morán Rosillo, Eric Mialhe, Benoit Diringer

**Affiliations:** 1 IncaBiotec SAC, Tumbes, Peru; 2 Tumbes National University, Tumbes, Peru,; 3 Buenaventura SA, San Isidro, Lima, Peru; 4 Xiamen Key Laboratory of Indoor Air and Health, Center for Excellence in Regional Atmospheric Environment, Key Lab of Urban Environment and Health, Institute of Urban Environment, Chinese Academy of Sciences, Xiamen, China; 5 Minera La Zanja SRL, San Isidro, Lima, Peru; 6 Concepto Azul SA, Estero Salado, Guayaquil, Ecuador; Universidade de Coimbra, PORTUGAL

## Abstract

Mine closure operations aim to restore the ecosystem to a near-original state. Microorganisms are indispensable for soil equilibrium and restoration. Metabarcoding was employed to characterize the bacterial and fungal composition in pristine soils, stockpiled soils (topsoils), enriched stockpiled soils (technosoils), enriched and revegetated soils (revegetated technosoils), and pit ecosystems in an open pit gold mine. Chao1 analysis revealed highest richness in pristine and topsoils, followed by technosoils (-17.5%) and pits (-63%). Bacterial diversity surpassed fungal diversity (-40%) in soil samples, but fungal OTUs were more abundant in pit samples (+73.4%). The findings identified the dominant microbial communities and conducted a comparative analysis of the shared microbiota. Dominant genera differed notably between pristine, topsoil, and technosoil samples for bacteria and fungi. The ecological indices’ results indicated that the pristine soil microbial communities were distinct from those in the topsoils, revealing significant alterations during the stockpiling process. The revegetated technosoil showed more similarity to the pristine and topsoil samples than to the freshly prepared technosoil, suggesting that microbial restoration is an ongoing phenomenon. Microbial restoration analysis revealed that Bacterial communities recover faster than fungal communities highlighting the potential of managing technosoil physicochemical parameters to enhance microbial recovery similar to those found in pristine soils. Runoff water contribute to this rebalancing by transporting microorganisms between ecosystem. All pit samples exhibited significant differences in their microbial composition, with moisture and rock composition representing the primary axes of dissimilarity. The greater community complexity observed in soils is related to the availability of nutrients, physicochemical variations, and the possibility of interaction with other microbes. Pits represent extreme ecosystems that limit the growth of most microorganisms. The presented research provides a scientific basis for future restoration strategies to improve microbial diversity and ecosystem resilience in altered landscapes.

## Introduction

Open-pit mining activities are surface-based exploitations that initially involve soil’s mechanical or explosive removal to access the mineral-bearing rocks [1]. This process displaces surface soils or topsoils, which are often stockpiled for extended periods, leading to alterations in their physicochemical properties [2]. New landscape structures, such as pits, roadways, and backfills, are also created, resulting in significant environmental impacts at biotic and abiotic levels [3].

Mine closure aims to restore the ecosystem and landscape to its original state. However, the reuse of topsoils presents significant challenges due to their extensive alterations, which hinder plant growth and soil recovery compared to pristine conditions [4]. Soil amendments can enrich topsoil, improve sediment conditions, promote plant growth, and restore ecological functions. Nevertheless, these engineered soils or technosoils often come with high costs, potentially compromising their long-term sustainability [5].

Soil microbial communities, especially fungi and bacteria, are central to ecosystem functioning by enhancing biogeochemical cycling, soil structure, contaminant breakdown, and symbiotic relationships with plants and animals. Balancing these communities is critical to the long-term success of soil and ecosystem restoration success [6–8]. However, the nearby presence of pits can negatively impact soil recovery due to the constant leakage of acidic water and its associated acid-forming microbiota [9,10]. Given these challenges, studying soil and pit microbiota is essential for sustainable soil remediation. Chronosequence studies, which assess ecosystem recovery over time, could provide valuable insights into microbiota patterns and inform strategies for sustainable soil restoration in mining-impacted areas [11–13]. Metabarcoding data obtained by high-throughput sequencing of environmental samples provide deep and comprehensive insights into the microbial biodiversity of the studied environment [14]. Its application is widespread in soil remediation contexts [15–17].

In this study, a metabarcoding approach was employed to identify both bacterial and fungal communities across soils and pit from an open-pit gold mine. Bioinformatic and statistical analyses were rigorously conducted to correlate the microbial structure with the physicochemical properties of these ecosystems during the operational and restoration phases in a mine located in the Cajamarca region, Peru.

This research represents a comprehensive effort to perform a comparative analysis of microbial community similarity within the context of soil recovery processes impacted by mining activities. Determining the composition of microorganisms in soils and pits will also facilitate future applications, such as 1) Optimizing soil amendment management to design technosoils that promote both rapid vegetation recovery and the development of specific microbial communities with essential ecological functions. 2) Identifying key microorganisms that serve as bioindicators of microbial recovery progress, enabling efficient monitoring of restoration efforts. 3) Selecting cultivable microorganisms of interest, such as those specialized in organic matter decomposition or nutrient cycling, to accelerate specific processes in soil restoration. 4) Promoting the use of biofilm-forming microorganisms to create microbial films on pit walls, reducing contaminant leaching and minimizing acid water production.

## Materials and methods

### Sample collection

**Fig 1:**
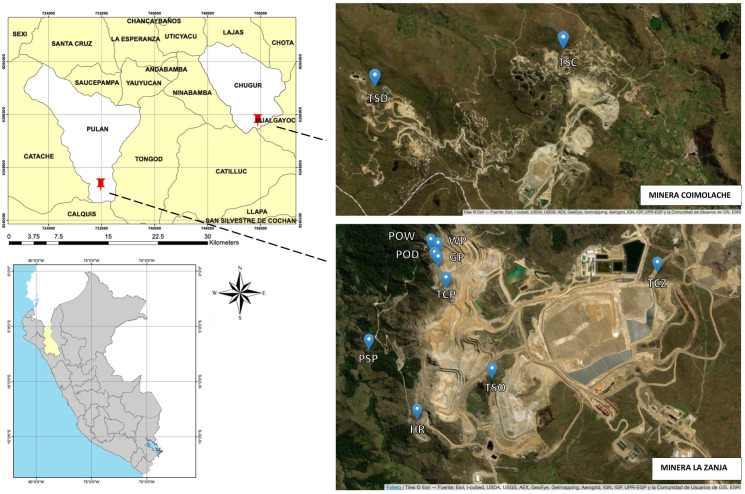
Geographic location of sampling points at La Zanja and Coimolache mining operations. The map was created from the free geoserver https://earthexplorer.usgs.gov reprinted from Geogpsperu under a CC BY license with permission from Geogpsperu, original copyright year 2024, and edited with ArcGis 10.7.1 version 2019.

Samples were collected during the dry season in August 2021 from Minera La Zanja S.R.L. (MLZ) and Compañía Minera Coimolache S.A. (CMC), both located in the department of Cajamarca, Peru (Fig 1). Samples were collected in and around the mining operations: Pristine soil (PSP) located out of MLZ, displaced and stockpiled soils (topsoil) from the DMO deposit at MLZ (TSO), topsoil from the Coimolache deposit (TSC), topsoil from Deposit 1 at CMC (TSD), recently amended topsoil (technosoil) prepared from MLZ topsoil (TCZ) and eight-month-old amended technosoil disposed and seeded with *Lolium sp., Trifolium sp., Festuca sp., Vicia sp., Lupinus sp.*, and *Cytisus sp*. (TCP). All sampling points are georeferenced in S1 Table. At each collection point, five sub-samples were recovered, and from these five sub-samples, a 1 kg sample was obtained as a homogenized composite. Sampling considered the guidelines proposed by the General Directorate of Environmental Quality of the Ministry of Environment [18].

Pit samples were collected from visually distinct pits using sterile swabs on 0.25 m^2^ of the pit in MLZ and preserved in BG11 liquid medium. Dry “orange” pit (POD), wet “orange” pit corresponding to wet pit due to water filtration (POW), water collected from the pit (WP), dry “gray” pit (GP), and hold road, the pit adjacent to the road (HR) were sampled. However, no samples were collected from pits in the Coimolache sector because rainfall began on the day of sampling, altering the conditions compared to the other samples.

Samples were kept at 4°C until reaching the laboratory and stored at -20°C until analysis. Additionally, physicochemical analyses of soils and pits were carried out to measure pH [19], Total Organic Carbon [20], texture [21], and Total Nitrogen [22]. To determine the lithology of the POW, POD, GP, and HR rock samples, Petrominerography and Petrology studies were carried out [23–26].

### Metabarcoding characterization

Total DNA extraction was performed using the Power Soil DNA Isolation Kit (MOBIO Laboratories Inc., Carlsbad, CA, USA) according to the manufacturer’s guidelines, which recommend using 0.25 g of sample. Metabarcodes were generated by PCR amplifying the V4 hypervariable region of the 16S rRNA gene using the 515F/806R primers for bacteria [27]. For fungi, the ITS1 and ITS2 variable regions were amplified using the ITS1F/ITS2R primers. In brief, total DNA was amplified with specific PCR primers in a 30-cycle one-step PCR using the HotStar Taq Plus Master Mix kit (Qiagen, USA) under the following conditions: 94°C for 3 min, followed by 28 cycles (5 cycles used for PCR products) of 94°C for 30 s, 53°C for 40 s and 72°C for 1 min, after which a final elongation step was performed at 72°C for 5 min.

Sequencing was performed at MRDNA on an Illumina MiSeq sequencer, and metabarcode data were processed using proprietary software analysis (MR DNA, Shallowater, TX, USA). Briefly, barcodes and primers were removed from sequences smaller than 150 bp, and sequences with ambiguous base-calling and homopolymer Runs exceeding 6 bp were removed. Operational taxonomic units (OTUs) were defined by clustering at 3% divergence (97% similarity) and removing unique sequences and chimeras. Final OTUs were taxonomically classified using BLASTn against a selected database derived from NCBI (www.ncbi.nlm.nih.gov).

### Calculation of ecological diversity and community similarity

Diversity indices and Pearson’s correlations were calculated using the Past4 program and R-Studio. Data validity was verified through manual calculations in Microsoft Excel 2021, treating the reads as the basis for these calculations. Additionally, Pearson’s correlation was confirmed using GraphPad Prism 10. BiPlot graphs, along with representations of Shannon’s index, Simpson’s index, inverse Simpson’s index, and Chao1, were produced in GraphPad Prism 10, as were the abundance and relative percent abundance of classes and genera. Violin plots were created for the “other” group among genera, and a two-way ANOVA analysis was conducted in GraphPad Prism 10. Canonical Correspondence Analysis (CCA) was performed using Past4 program to assess the relationship between species abundance and environmental variables, including pH, Organic Carbon content, and total nitrogen content.

### Ethical aspects

This study including sampling and use of *Lolium sp., Trifolium sp., Festuca sp., Vicia sp., Lupinus sp.,* and *Cytisus sp*. were approved by the National Forest and Wildlife Service (SERFOR), registered under license RDG No. D000237-2021-MIDAGRI-SERFOR-DGGSPFFS. All seeds used were commercially available and purchased in Cajamarca City.

## Results

### Sampling

Soil and rock properties were determined (S1 Table). The sampled soils tended to be acidic, with a pH ranging from 3.9 to 4.7, while the technosoil had a pH close to neutral (6.5 to 7.7). Adding amendments to the technosoils gives them a sandy loam texture, while the topsoils and pristine soils are clay loam soils. The soils had organic carbon contents ranging from 3 to 8.8%. The Coimolache topsoils had lower total nitrogen concentrations than the La Zanja mine area. Among the pits, only the Hold Road (HR) has a pH of 4.5, similar to pristine soils, while the other open-pit types are highly acidic (pH:1-2). The HR pits have an outer layer corresponding to a combination of rock, soil, and vegetation (Bryophyta, Pteridophyta, Spermatophyta), while the orange and gray pits were composed of bare rock. HR had the highest total carbon content and the lowest sulfur concentration. Grey pit (GR) had the highest sulfur concentration before Dry and Wet Orange pit (POD, POW), while iron content was similar between all pit samples.

### Sequencing data

The sequencing process of region V4 generated 382,766 raw sequences (S2 and S3 Tables). After stringent filtration, 293,123 high-quality curated sequences (75.8%) were obtained [28, 29]. ITS1-2 sequencing yielded 668,176 raw sequences, resulting in 338,165 curated sequences (50.6%) after filtration. Most filtered sequences were from topsoil TSD, TSC, TSO, and WP samples, identifying 2,832 bacterial OTUs and 1,519 fungal OTUs (>97% identity threshold).

Ecological diversity indices like Shannon and Chao-1 were used to compare communities and assess changes in biodiversity (S2 and S3 Tables). Soil samples generally had higher richness indices than pit samples, except for water collected from the pit (WP), likely due to soil leaching. The gray pit (GP) sample exhibited the lowest diversity, suggesting a less diverse environment. Bacterial communities were often more varied than fungal communities in soils but not in pits.

### Metabarcoding of soil- Bacteria

The study conducted metabarcoding of soil bacteria, comparing microbiota in Pristine Soil (PSP), topsoil storage areas (TSD, TSO, TSC), and the technosoils (TCZ, TCP).

Bacterial biodiversity across these samples included 23 distinct phyla (mean 19.3), 56 classes (mean 44.8), and 415 genera (mean 251.2). Technosoils (TCZ, TCP) had fewer phyla but higher genus diversity, while pristine soil had the lowest genus number (S4 Table).

**Fig 2:**
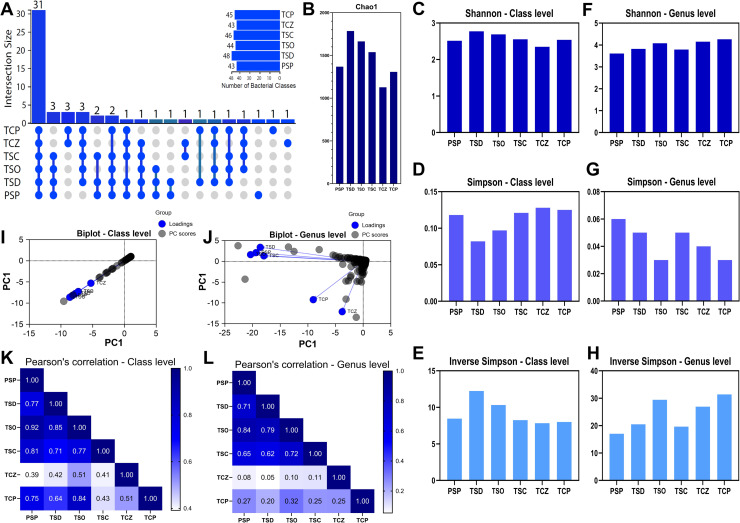
Taxonomic diversity of Prokaryotes in soil samples. **A:** UpSet Plot for bacterial classes showing overlap between samples, **B:** Chao1 Index at OTUs level, **C:** Shannon index at class level, **D:** Simpson index at class level, **E:** Inverse Simpson’s index at class level, **F:** Shannon index at genus level, **G:** Simpson index at genus level, **H:** Inverse Simpson’s index at class level, **I**: Biplot at class level, PC1 represent the abundance data (RPKM), **J:** Biplot at the genus level, PC1 represent the abundance data (RPKM), **K**: Pearson correlation at the class level, **L:** Pearson correlation at the genus level. PSP: Pristine soil, TSD; topsoil from Deposit 1, TSO; topsoil from the DMO deposit, TSC; topsoil from the Coimolache deposit, TCZ; recently amended topsoil (technosoil), TCP; eight-month-old amended technosoil.

The UpSetPlot analysis shows that 31 out of 56 (55.4%) bacterial classes were common to all samples, suggesting the existence of a bacterial core common to all soil types analyzed (Fig 2A). The most frequent class overlaps were between TCZ and TCP, PSP and topsoils (TSO, TSD, TSC), and all samples except PSP. Some classes were unique to PSP, TCZ, and TCP.

The values of the Chao1 analysis reflect variability in the estimation of species richness among the different samples analyzed. Topsoil samples (TSD, TSO, and TSC) presented the highest OTUs richness, followed by pristine soil (PSP) and technosoil (TCP, TCZ) (Fig 2B). These data suggest differences in potential biodiversity among the samples.

At the class level, the Shannon index, which measures diversity considering the taxa richness and evenness, shows that TSD has the highest diversity, while TCZ has the lowest value (Fig 2C). For Simpson’s index, which reflects the probability that two randomly selected individuals are of the same species, the analysis indicates that the TSD sample has the lowest species dominance, suggesting higher equity than other samples (Fig 2D). Inverse Simpson’s index, which measures diversity as a function of the number of dominant species, shows that TSD has the highest effective diversity, while TCZ has the lowest (Fig 2E). These indices suggest that the topsoil TSD, followed by TSO and TSC, have higher diversity and equity than the other samples. In comparison, TCZ has lower diversity and equity. Soils that present vegetation, PSP and TCP, show similar levels of diversity richness.

At the genus level, TCP, TCZ, and TSO have the highest Shannon index values (Fig 2F), indicating the highest diversity and equity among the samples, while PSP has the lowest value, probably due to the heterogeneity of the species. Species dominance revealed by Simpson’s index shows that TSO and TCP have the lowest values, suggesting a lower dominance and more even species distribution in these samples (Fig 2G). Inverse Simpson’s index indicates that TCP has the highest value, suggesting a higher effective diversity, while PSP has the lowest value (Fig 2H). At the genus level, the results indicate that TCP has the highest overall diversity and equity, followed by TSO and TCZ. In contrast, PSP has the lowest values in all diversity indices analyzed. Observing these variable results between class and genus, we could argue that there is a greater diversity of species in the classes present in TCZ compared to the other samples.

At the class level, the biplot dispersion analysis showed a high interrelationship in taxonomic composition between samples. Sample TCZ was slightly away from the “cluster” without disturbing the trend (Fig 2I). At the genus level, a dispersion of data in the opposite direction to the principal component is observed, with a clustering pattern showing the TCZ point with the lowest interrelation with the others. However, TCP is close to TCZ, and their axe angle suggests a low correlation between them but higher than with the other samples. The arrangement between the other points (natural soils PSP, TSO, TSC, TSD) suggests a relevant similarity (Fig 2J).

By analyzing Pearson’s correlation at the class level, it was possible to observe that PSP, TSD, TSC, and TSO are highly correlated (Fig 2K), confirming similarities in their bacterial communities. TCP has generally lower correlation indices, especially with TSC and TCZ. On the other hand, TCZ lacked correlation with the rest of the samples. In turn, correlation at the genus level highlights that TCP and TCZ show higher dissimilarities with natural soils PSP, TSO, TSD, and TSC, following a correlation pattern similar to that of class (Fig 2L).

**Fig 3:**
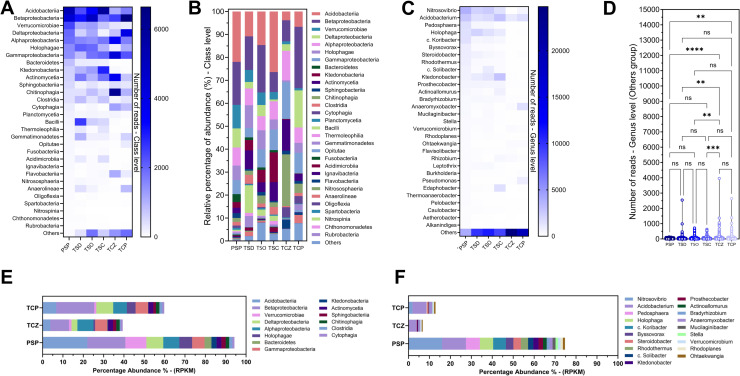
Taxonomic composition of Prokaryotes in soil samples **. A:** Abundance of reads at the class level, **B:** Relative percentage abundance at the class level, **C:** Abundance of reads at the genus level, **D:** Violin plot for the “Others” group, two-way ANOVA differencing test. **E:** Comparison of main bacterial class prevalence (>1%) between pristine and technified soils during restoration, **F:** Comparison of main bacterial genus prevalence (>1%) between pristine and technified soils during restoration. PSP: Pristine soil, TSD; topsoil from Deposit 1, TSO; topsoil from the DMO deposit, TSC; topsoil from the Coimolache deposit, TCZ; recently amended topsoil (technosoil), TCP; eight-month-old amended technosoil.

Significant variations were observed in the abundance of different bacterial classes among samples (Fig 3A and 3B). Acidobacteria predominated in PSP and TSC, while Betaproteobacteria were more abundant in TCP and TSO. Verrucomicrobiae was prominent in PSP and less prevalent in TCZ. Deltaproteobacteria showed its highest abundance in TCP, while Alphaproteobacteria had high levels in TCZ. Classes such as Bacteroidetes, Ktedonobacteria, and Actinomycetia showed remarkable variations in their distribution. We can also mention that Chitinophagia reached high levels in TCZ. The less abundant classes categorized in a group as “Others” were shown to be more abundant in TCP, TSO, and TCZ, suggesting that these samples have more bacterial classes capable of contributing to the functionality of the microbiome present. These results reflect considerable diversity in bacterial composition among the samples analyzed.

Among the most prominent genera (Fig 3C), *Acidobacterium* was the only genus with more than 1% prevalence in all samples, although its presence in TCZ was reduced. High prevalence of *Nitrosovibrio* and *Holophaga* (average 10.9% and 6.7%) were detected in “natural soil samples”, in particular, pristine soil PSP and Topsoil from the DMO1 TSD sample. In comparison, its abundance decreases notably in technosoils (average 1.0% and 0.1%). Another relevant genus is *Pedosphaera*, which is predominantly found in PSP, while its presence decreases in the other samples. Other more discrete genera were also found shared among topsoils with pristine soil and almost absent in technosoils such as *Pedospharea*, *Candidatus koribacter*, *Steroidobacter*, *Rhodothermus*, *Candidatus solibacter*, *Prosthecobacter*, *Bradyrhizobium*. The most prominent and common genera among the topsoils were *Ktedonobacter*, *Thermoflavimicrobium*, and *Methylobacter*. The genera Bacteroides and *Acidovorax* were common in technosoils (averages 3.0% and 2.9%) and presented low prevalence in natural soils (0.3% and > 0.1%). The genus *Flavihumibacter* (14.6%) was highly represented in the TCZ sample and non-existent in almost all other samples. The TCP sample showed the highest diversity of predominant genera such as *Thiobacillus*, *Anaeromyxobacter*, *Geobacter*, *Geothrix*, and *Pseudomonas*.

The “Others” analysis includes various genera with reads that collectively exceed those most abundant in almost all samples (Fig 3D). The grouping of the thirty most abundant genera, with reference to PSP and the taxonomic constitution in the “Other” group, could perhaps reference the “bacterial core”. Two-way ANOVA analysis showed a significant difference in TCZ with all other samples except with TCP. Significant differences were also observed in the TCP-PSP comparison. The highest difference was between TCZ-PSP. These results support the above analyses, placing TCZ and TCP as the most dissimilar samples from the rest.

The analysis of bacterial classes with a prevalence greater than 1% indicates that the similarity to the pristine soil (PSP) community increases from 39.6% in the freshly prepared technosoil (TCZ) to 59.8% in technosoil after eight months of revegetation (TCP) (Fig 3E and S5 Table). Key classes such as Acidobacteria, Deltaproteobacteria, Betaproteobacteria, and Holophaga show positive recovery, with Betaproteobacteria and Deltaproteobacteria exceeding PSP levels. In contrast, Alphaproteobacteria, Gammaproteobacteria, and Actinomycetia display a reduction in prevalence from TCZ to TCP, reaching levels closer to those in PSP, indicating stabilization within the bacterial community. Similarly, classes such as Cytophagia, Chitinophagia, and Actinomycetia, which had high initial prevalences in TCZ, show significant reduction in TCP, but remain higher than in PSP. Classes such as Bacteroidetes, Ktedonobacteria, and Verrucomicrobia show the lowest recovery rates in TCP, suggesting that longer recovery times are needed to reach PSP levels. At the genera level, the community similarity to PSP increases from 7% in TCZ to 13% in TCP (Fig 3E and S6 Table). Genera such as *Acidobacterium* and *Ohtaekwangia* demonstrate notable recovery, reaching 58.1% and 70.8% of PSP levels, respectively. Additionally, *Anaeromyxobacter* shows significant proliferation, increasing from 1.4% in PSP to 6.1% in TCP (434.7% recovery), suggesting a role in early soil restoration. Conversely, key genera such as *Nitrosovibrio*, *Pedosphaera*, and *Holophaga* show significant increases in TCP compared to TCZ but remain low compared to PSP, implying longer recovery times.

Canonical Correspondence Analysis (CCA) is an ordination method used to explore relationships between species composition and environmental variables, providing insight into how specific variables influence the distribution of microbial communities. In our study, CCA was performed to evaluate the influence of key soil variables, pH, organic carbon (C.O.), and total nitrogen (T.N), on bacterial composition in pristine soils (PSP), topsoils (TSD, TSC, TSO), and technosoils (TCZ and TCP) (S1 Fig. y S2 Fig.). The CCA results positioned bacterial classes and genera along gradients of these variables, allowing us to identify potential drivers of community shifts. For example, to promote the recovery of Verrucomicrobiae while reducing the prevalence of Chitinophagia in TCZ and TCP, pH would need to be lowered while organic carbon levels were significantly increased. Similarly, to favor Acidobacteria recovery in technosoils, both a decrease in pH and an increase in organic carbon would be required, along with a slight increase in total nitrogen. At the genus level, *Nitrosovibrio* recovery in TCP would require a significant decrease in pH, a large increase in total nitrogen, and a decrease in organic carbon.

In summary, these detailed data reflect significant variations in the diversity, dominance, and composition of bacterial communities in soils with different environmental conditions. Altogether, the differences in diversity indices and multivariate and relative abundance analyses show that the native soil samples (PSP, TSC, TSD, TSO) formed a cluster separated from the two technosoil samples that shared more remarkable similarities. Among the soil samples, the DMO topsoil and DM1 topsoil (TSO-TSD) were the most similar, separating from the Coimolache soils (TSC) and the pristine soil.

### Metabarcoding of pit – Bacteria

The excavations necessary to extract the minerals generate pits that may present different particularities depending on their mineral composition. In this case, the microbiota on the pits adjacent to the revegetated technosoil of the San Pedro area (TCP) was evaluated. These pits were separated as follows: gray pit (PG), dry orange pit (POD), wet orange pit (POW), and runoff water collected in the San Pedro pit (POW). A pit adjacent to the Hold Road (HR) of the La Zanja mining operation was also collected.

The microbial diversity of the pits represented only 40% of that of the soils, with a total of 13 distinct phyla (mean 8) and 30 bacterial classes (mean 14.4). The water sample (WP) and the Hold Road sample (HR) had, respectively, the highest (23) and lowest (9) number of classes. A total of 169 bacterial genera were identified (mean 58.4). The highest number of genera was found in the orange wet well, with 73 genera, and the lowest in the Hold Road, with 45 genera (S4 Table).

**Fig 4:**
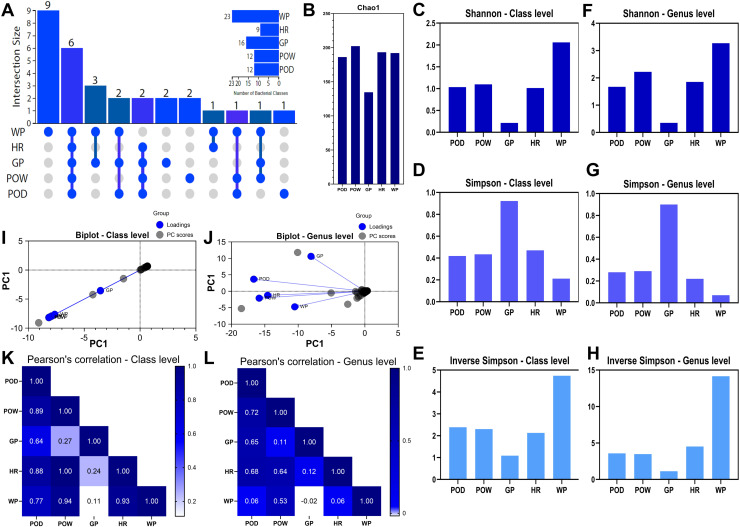
Taxonomic diversity of Prokaryotes in pits samples **. A:** UpSet Plot for bacterial classes showing overlap between samples, **B:** Chao1 index at OTUs level, **C:** Shannon index at class level, **D:** Simpson index at class level, **E:** Inverse Simpson’s index at class level, **F:** Shannon index at genus level, **G:** Simpson index at genus level, **H:** Inverse Simpson’s index at class level, **I:** Biplot at class level, PC1 represent the abundance data (RPKM), **J:** Biplot at the genus level, PC1 represent the abundance data (RPKM), **K:** Pearson correlation at the class level, **L:** Pearson correlation at the genus level. POD; Dry orange pit, POW; wet orange pit, GP; dry gray pit, HR; hold road, WP; water collected from the pit.

The UpSet plot analysis of the bacterial classes showed that 30% of the classes identified were specific to the effluent (WP) (Fig 4A). On the contrary, 20% of them were common to all samples. Subsequent intersections identified three classes (10%) that overlapped between the gray wall GP and WP. Interestingly, only seven classes were shared between WP and GP despite direct contact.

The measurement of total species richness estimated by the Chao1 index reflects a higher richness of bacteria in POW, HR, WP, and POD compared to GP, suggesting higher diversities in those samples (Fig 4B). Notably, the values reached by Chao1 analysis in bacteria from pits are considerably lower than those obtained in soils, indicating, a priori, a lower total specific richness despite having a higher number of reads in pits (S2 Table).

The Shannon index measures species diversity with emphasis on their evenness. In this regard, it was found that the effluent water (WP) and, to a lesser extent, the POD, POW, and HR pits contain significant uniform richness at both the class and genus levels compared to the grey pit GP (Fig 4C and 4F). This pattern is repeated in the inverse Simpson’s analysis, where the bacterial community in GP presents much fewer rare species than in the other pits, especially in the WP water (Fig 4E and 4H). This observation is even more pronounced at the genus level. Simpson’s analysis, which is more sensitive to common species, estimated its highest value in GP and its lowest value in WP, both at the class and genus level (Fig 4D and 4G).

Principal component analysis at the class level showed a high interrelationship (correlation or similarity) between samples at the taxonomic composition level. However, the GP sample was slightly separated from the “cluster” without breaking the trend (Fig 4I). At the genus level, a scattering of the data is observed, aligned in the same direction as the principal component, with a clustering pattern showing the GP point with the least interrelationship with the others, although POD is somewhat close to GP. However, the angle of the axes suggests a low correlation between them, but higher than with the other samples. The arrangement of the other points suggests a significant interrelationship (correlation or similarity) between them, with a higher degree of correlation between POW and HR (Fig 4J).

Along with this analysis, Pearson’s correlation index at the class level confirmed the low similarity of Grey Pit GP with the other samples, with the dry orange pit POD being the most closely related (Fig 4K). However, the POD sample showed a higher similarity between POW and HR. The POW sample obtained perfect similarity with HR and a very close structure relationship with WP runoff water. At the genus level, POD was the sample with the best similarities, particularly with POW, HR, and GP (Fig 4L). Surprisingly, the correlation indices between WP and the other samples were drastically reduced at the genus level.

**Fig 5:**
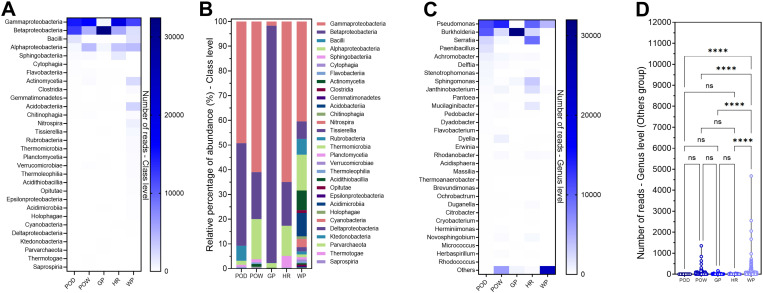
Taxonomic composition of Prokaryotes in pits samples. **A:** Abundance of reads at the class level, **B:** Relative percentage abundance at the class level, **C:** Abundance of reads at the genus level, **D:** Violin plot for the “Others” group, two-way ANOVA differencing test. POD; Dry orange pit, POW; wet orange pit, GP; dry gray pit, HR; hold road, WP; water collected from the pit.

Taxonomic assignment at the class level showed that Gammaproteobacteria were predominant in all samples except the GP gray wall (Fig 5A). In turn, Betaproteobacteria were strongly represented in all samples, with a high dominance in GP (96.0%) and POD (41.5%) (Fig 5B). Also noteworthy is the prevalence of Alphaproteobacteria in POW, HR, and WP. These three classes harbor the core microbiota of the walls. Notably, the runoff water presented a greater diversity of bacterial taxa, especially of low prevalence, as shown by the importance of the “Other” cluster (Fig 5B and Fig 5C).

At the genus level, the pit microbiota was dominated by *Burkholderia* (average 29.3%) and *Pseudomonas* (average 27.5%), followed by *Serratia* (average 7.7%) (Fig 5C). *Pseudomonas* was the predominant genus in POD, POW, and HR pits samples, while *Burkholderia* comprised 94.6% of the community in the gray pits. In the containment road pits HR, the genus *Serratia* reached 30.2%. Also note the presence of *Sphingomonas*, *Janthinobacterium*, and *Mucilaginibacter*. With the notable presence of *Pseudomonas* and the near absence of *Burkholderia*, the microbiota presents in the runoff water proved to be significantly more diverse than in the other samples (Fig 5D).

Bacterial populations collected from pits adjacent to revegetated technosoils generally showed significantly lower taxonomic compositions and diversity indices than those found in soils. The most remarkable similarities between these ecosystems were found between the Wet Orange Pit and the Hold Road Pit (POW-HR), followed by the Dry Orange Pit (POD). The Gray Pit (GP) sample moves away from this group due to its low microbial diversity, which *Burkholderia* strongly dominates. This distribution does not match the characteristics and composition of the rocks that make up these pits: strong acidity in POW, POD, and GP, high (GP), moderate (POD/POW), and almost no sulfide levels (HR), and different compositions at the mineralogical level. The runoff water sample was found to be very distant due to the higher microbial diversity it carries, probably acquired by passing through different ecosystems.

### Metabarcoding of soil- fungi

For fungi, samples showed 7 to 9 phyla, totaling 11 phyla, 35 different classes were observed, with TSC and TSO having the lowest diversity (26) and TSD the most (30) for an average of 27.5. Finally, a total of 368 genera were found (average 213.3). In general, technosoils had lower fungal diversity (S4 Table).

**Fig 6:**
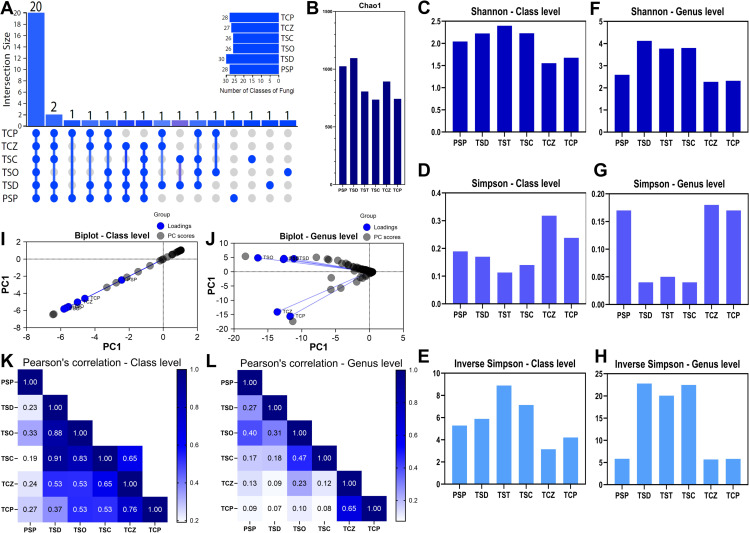
Taxonomic diversity of fungi in soil samples. **A:** UpSet Plot for fungal classes showing overlap between samples, **B:** Chao1 index at OTUs level, **C:** Shannon index at class level, **D:** Simpson index at class level, **E:** Inverse Simpson’s index at class level, **F:** Shannon index at genus level, **G:** Simpson index at genus level, **H:** Inverse Simpson’s index at class level, **I:** Biplot at class level, PC1 represent the abundance data (RPKM), **J:** Biplot at the genus level, PC1 represent the abundance data (RPKM), **K:** Pearson correlation at the class level, **L:** Pearson correlation at the genus level. PSP: Pristine soil, TSD; topsoil from Deposit 1, TSO; topsoil from the DMO deposit, TSC; topsoil from the Coimolache deposit, TCZ; recently amended topsoil (technosoil), TCP; eight-month-old amended technosoil.

The Upset Plot analysis shows that most fungal classes (57.1%) were present in all soil types analyzed (Fig 6A). The other class overlaps did not show a clear pattern indicating ecological similarities. Nevertheless, PSP and TCP exhibited nine different class interactions with other samples, whereas TSC presented only six. Some classes were found to be unique to PSP, TSO, TSD, and TSC.

Analysis of the Chao1 index indicates that TSD and PSP had the highest species the soil samples (Fig 6B). In contrast, TSC, TCP, and TSO had the lowest. The Chao1 indices of soil fungi were lower than those of bacterial diversity.

At the class level, Shannon indices were notably higher in TSO, TSC, TSD, and PSP compared to reconstructed soils (TCZ and TCP), indicating higher fungal species richness and evenness in natural soils (Fig 6C). An inverse trend was observed for Simpson’s index, suggesting a more equitable species distribution in the technosoils (Fig 6D).

The inverse Simpson’s index analysis shows a clear superiority of TSO in terms of effective diversity, followed by TSD, TSC, and PSP. On the contrary, the TCZ sample has the lowest value. The saprophytic character of most of the fungi could explain their higher prevalence in the stockpiled soils, which correspond to highly altered soils, compared to PSP, TCP, or TCZ soils.

At the genus level, the Shannon and inverse Simpson’s indices of the topsoils (TSD, TSO, and TSC) were significantly higher than those of the other samples (Fig 6F and 6G). Simpson’s indices were higher in TCZ, PSP, and TCP, although they were relatively similar (Fig 6G). Similarly, Simpson’s TSD, TSC, and TSO indices were equivalents but much lower than the previous ones.

Principal component analysis (PCA), plotted in BiPlot, showed a high interrelationship (correlation or similarity) of taxonomic composition between samples at the class level. PSP slightly deviates from “group” without disrupting the trend (Fig 6I). At the genus level, a scattering of data is observed that is oriented in the opposite direction of the principal component, with a clustering pattern that shows the TCP point with the least relatedness to the others (Fig 6J). Although the proximity of TCZ to TCP indicates some similarity between the technosoils, the angle of the axes suggests a low correlation between them but higher than with the other samples. The arrangement between the different points suggests a significant interrelationship (correlation or similarity) between the “natural” soils.

The Pearson correlation plot at the class level clearly shows the low correlation of PSP with all other samples (Fig 6K). On the contrary, the high correlations between the 03 topsoil samples (TSD, TSC, TSO) reveal a very similar fungal class composition. The technosoil samples showed high correlations with each other and moderate similarities with the topsoil samples.

At the genus level, the highest similarities were observed between TCP and TCZ, followed by TSC with TSO and TSO with PSP, although these correlations were moderate (Fig 6L). The remaining correlations between samples were low, indicating their community composition is very different.

**Fig 7:**
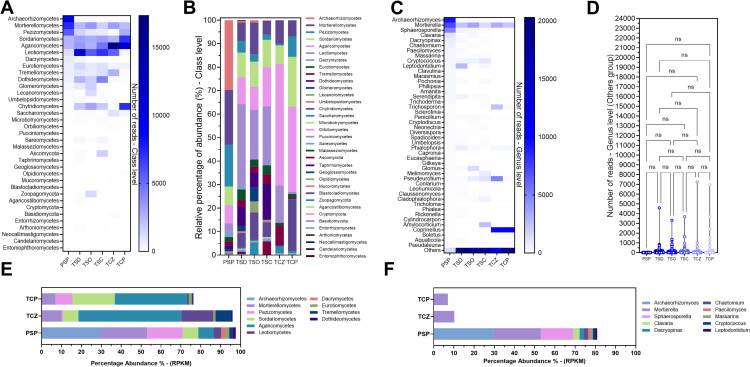
Taxonomic composition of fungi in soil samples. **A:** Abundance of reads at the class level, **B:** Relative percentage abundance at the class level, **C:** Abundance of reads at the genus level, **D:** Violin plot for the “Others” group, two-way ANOVA differencing test. **E:** Comparison of main fungi class prevalence (>1%) between pristine and technified soils during restoration, **F:** Comparison of main fungi genus prevalence (>1%) between pristine and technified soils during restoration. PSP: Pristine soil, TSD; topsoil from Deposit 1, TSO; topsoil from the DMO deposit, TSC; topsoil from the Coimolache deposit, TCZ; recently amended topsoil (technosoil), TCP; eight-month-old amended technosoil.

The heatmap representation of the distribution of fungal classes highlights the importance of Mortierellomycetes, Sordariomycetes, and Agaricomycetes (mean 10.4%, 10.6%, 21.1%, respectively) as a microbiological core (Fig 7A and 7B). Other classes, such as Pezizomycetes and Leotiomycetes, are abundant in most soil samples. Therefore, the predominance and specificity of Archaeorhizomycetes in the pristine soil is remarkable since it represents up to 28.3% of the total abundance, while it is almost absent in the other samples. It is also important to note the relative importance of Agaricomycetes in the freshly prepared techonosol (51.5%). Finally, several “secondary” classes such as Eurotiomycetes, Tremellomycetes, Dothideomycetes, and Chytridiomycetes are mainly found in the topsoils, although Chytridiomycetes was also abundant in the TCP.

At the genus level, the Pristine soil PSP is the only sample with several dominant genera, the most important being *Archaeorhizomyces*, *Mortierella*, and *Sphaerosporella* (Fig 7C). *Mortierella* was the most common genus among all the samples (with a mean of 11.2%) and was also the only one present in percentages higher than 1% in each soil type. Apart from sporadic occurrences of *Leptodontidium* in TSD or *Trichosporon* in TCZ, the topsoil and technosoil samples showed fewer common genera grouped as “others”. Interestingly, the two-way ANOVA differencing test showed that these groups corresponding to “others” were not significantly different from each other, indicating high taxonomic complexity (Fig 7D).

The analysis of fungal classes with > 1% prevalence in PSP reveals limited recovery, with community similarity increasing slightly from 33.3% in freshly prepared technosoil (TCZ) to 34.4% after eight months of revegetation (TCP) (Fig. 7E and S7 Table). Key PSP classes such as Archaeorhizomycetes showed no recovery, Mortierellomycetes decreased in similarity from TCZ to TCP while Pezizomycetes increased significantly. Sordariomycetes exhibited rapid proliferation, exceeding PSP levels in TCP by 267.2%, whereas Agaricomycetes showed partial recovery but remained higher than PSP, indicating ongoing stabilization. At the genus level, only 7.1% of genera in TCP were similar to those in PSP, representing a decline from the 10.3% similarity present in TCZ (Fig. 7F and S8 Table). This reduction was primarily driven by the decreased prevalence of *Mortierella* in TCP. Overall, no substantial changes were observed, suggesting that key fungal genera from PSP are not playing a dominant role in the soil recovery process.

Canonical Correspondence Analysis (CCA) performed on the fungal communities revealed that classes such as Archaeorhizomycetes, which are highly specific to PSP, would benefit from a significant decrease in pH and an increase in total nitrogen (T.N) and organic carbon (O.C.) (S3 Fig). In contrast, Agaricomycetes, which are prominent in the freshly prepared technosoil (TCZ) and in the technosoil after revegetation (TCP), seem to naturally stabilize around the values observed in PSP, suggesting a self-balancing trend (S7 Table). At the genus level, the recovery of *Archaeorhizomyces* would require careful balancing of pH, TN and O.C. to match PSP levels (S4 Fig). Meanwhile, a moderate decrease in pH combined with an increase in TN and C.O. could favor the proliferation of *Mortierella*, a genus widely distributed in all soil types but with a remarkably low prevalence in TCP. The strong prevalence of some classes and genera observed in pristine soils (PSP) suggests that additional environmental variables may need to be considered to fully explain their distribution patterns.

All ecological diversity indices of fungi in soils were generally lower than those observed for bacteria. However, it is emphasized that the fungal communities of topsoils and pristine soils show more remarkable ecological similarity to each other and form a separate group from the technosoil samples, as revealed in the case of bacterial communities.

### Metabarcoding of pit- fungi

Pit fungal barcode metacode analysis compared the microbiota in oxidized “orange” dry pit (POD), “orange” wet pit (POW), water collected from the pit (WP), “gray” dry pit (GP), and roadside pit (HR).

The fungal biodiversity in these samples includes nine different phyla (mean 7.4), 31 classes (mean 24.4), and 293 genera (mean 176.2). The samples are very similar regarding the number of phyla and classes, but the sample WP differs from the others, with 228 genera (S4 Table). Notably, the number of fungal classes and genera in pits is much higher than that of bacteria.

**Fig 8:**
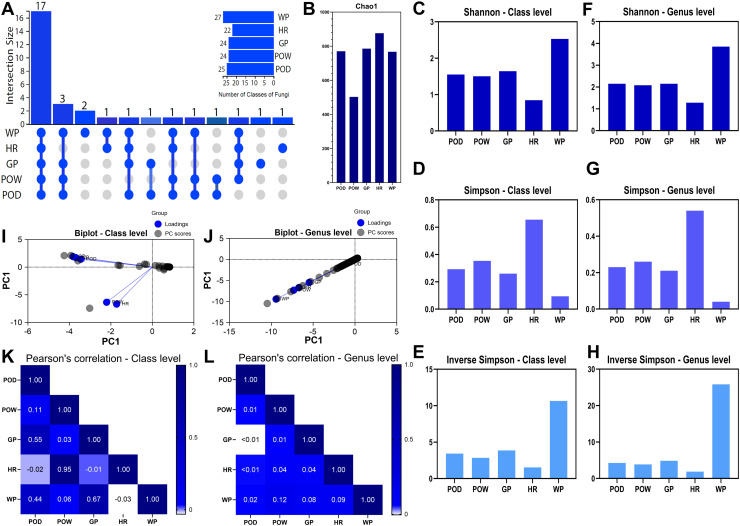
Taxonomic diversity of fungi in pit samples **. A:** UpSet Plot for fungal classes showing overlap between samples, **B:** Chao1 index at OTUs level, **C:** Shannon index at class level, **D:** Simpson index at class level, **E:** Inverse Simpson’s index at class level, **F:** Shannon index at genus level, **G:** Simpson index at genus level, **H:** Inverse Simpson’s index at class level, **I:** Biplot at class level, PC1 represent the abundance data (RPKM), **J:** Biplot at the genus level, PC1 represent the abundance data (RPKM), **K:** Pearson correlation at the class level, **L:** Pearson correlation at the genus level. POD; Dry orange pit, POW; wet orange pit, GP; dry gray pit, HR; hold road, WP; water collected from the pit.

Analysis of the altered plot shows that 17 of the 31 fungal classes (54.8%) were common to all samples, suggesting the existence of a core microbiome common to all pit types analyzed (Fig 8A). The next most common class overlap was between POD, POW, GP, and WP, excluding HR. Some classes were found exclusively in WP (6.5%), GP (3.2%), and HR (3.2%) samples.

The Chao1 analysis values reflect that the Hold Road (HR) sample presented a higher OTU richness, as it is a pit visually colonized by vascular plants, mosses, and lichens. However, in second place are the GP, POD, and WP samples, devoid of vegetation, with the wet pit (POW) containing the lowest diversity (Fig 8B).

The analysis of the Shannon index at the class level showed that the WP sample presented the highest diversity and uniformity of species. In contrast, the HR sample presented the lowest value (Fig 8C). The POD, POW, and GP samples showed intermediate values that were very close to each other. The indices showed a similar pattern at the genus level (Fig 8F).

Simpson’s index, it reflects that the HR sample harbors some classes and genera more dominant than others (Fig 8D and 8G). The POD, POW, and GP samples showed significantly lower values, with WP being the lowest. Inverse Simpson’s index indicates that WP has the highest effective diversity while HR has the lowest (Fig 8E). Overall, the diversity indices analyzed in the pits showed that the fungal communities exhibited greater richness and diversity than the bacterial communities at both class and genus levels.

Biplot analysis at the class level shows a dispersion of data in the opposite direction to the principal component, with a clustering pattern between HR and POW samples indicating some proximity. At the same time, the arrangement of POD, WP, and GP suggests a significant relationship (correlation or similarity) between them (Fig 8I). At the genus level, a high interrelationship (correlation or similarity) between the samples is observed to a greater extent between HR and POW (Fig 8J).

In the Pearson correlation analysis at the class level, it was observed that the POD sample is correlated with GP, followed by WP, and a lower correlation with the other samples. On the other hand, the POW sample is highly correlated with the HR sample. The GP sample correlates with the WP sample (Fig 8K). At the genus level, all samples show a very low correlation with each other (Fig 8L).

**Fig 9:**
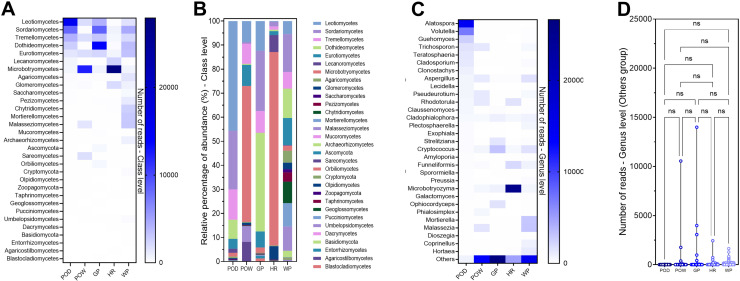
Taxonomic composition of fungi in pit samples **. A:** Abundance of reads at the class level, **B:** Relative percentage abundance at the class level, **C:** Abundance of reads at the genus level, **D:** Violin plot for the “Others” group, two-way ANOVA differencing test. POD; Dry orange pit, POW; wet orange pit, GP; dry gray pit, HR; hold road, WP; water collected from the pit.

Some significant differences in the abundance of fungal classes were found between samples. Leotiomycetes and Sordariomycetes predominate in the POD sample. Microbotryomycetes predominate in the POD and HR samples. Dothideomycetes and Sordariomycetes predominate in the GP sample, while a greater diversity of genera, without clear predominance, is observed in the WP sample (Fig 9A and 9B).

Among the most prominent fungal genera in the pits, *Alatospora*, *Volutella*, and *Guehomyces* are found in the POD sample, although their presence is deficient in the other samples. The prevalence of *Cryptococcus* was detected in the GP sample. In the HR sample, the genus *Mycrobotryozyma* predominates. On the other hand, in the WP sample, more discrete genera, such as *Mortierella* and *Malassezia*, were found in their dominance (Fig 9C). The “Others” cluster was significant in the POW, GP, and WP samples, with several reads that exceeded the most abundant genera. Two-way ANOVA analysis showed no significant differences between the “others” clusters (Fig 9D).

Overall, the data collected reflect significant variations in the diversity, dominance, and composition of fungal communities in pits compared to bacterial communities. However, both analyses show the richness of WP, which harbors a higher diversity of species not found in the other samples.

## Discussion

The assessment of microbial composition in mining environments is of growing interest because microorganisms are essential for the resilience of disturbed ecosystems [30–32]. Displacement and piling of soils and rocks exposed to weathering cause significant physicochemical changes that alter the composition and functionality of these communities [33]. This study aimed to comprehensively compare the microbial communities of different soil types and pit ecosystems in an open-pit gold mine.

A Chao1 index value above 100 or 200 can be considered to indicate relatively high sample richness [34]. All samples exhibited Chao1 values above 100 and some above 1000, suggesting the presence of numerous species not detected in the sampling. Generally, Chao1 indices were higher in the soil samples than in the pits for bacterial and fungal communities, probably due to the greater complexity of the soils in terms of nutrient availability, physicochemical variations, and the possibility of interaction with other microorganisms [35]. On the contrary, pyroclastic pits are considered extreme ecosystems; they are poor in nutrients, exposed to UV radiation, and to the formation of acidic waters that limit the growth of most microorganisms [36].

The Shannon index can measure species diversity in a community that has been disturbed, providing a numerical value that reflects the diversity of the community and is not affected by sample size [37]. A numerical value away from 0 implies a community rich in species that are evenly distributed in terms of relative abundance. In our study, all soil samples presented a high bacterial richness, superior to pits. In the case of the bacterial community in soils, Shannon indices were globally higher in natural soils at the class level but inferior at the genus level. This indicates that the creation of technosoils has caused simplification at the class level but diversification at the genus level. This trend suggests that human intervention in creating technosoils has significantly altered the microbial community, promoting genus-level diversification/selection within a narrow set of microbial classes. Short-term benefits on soil microbial functionality have been reported in MLZ, with the success of revegetation operations such as in the TCP area compared to failed attempts to use “raw” topsoils for direct soil revegetation. Pit samples had a lower value than soils for both communities. In pits, Shannon indices were generally higher for fungi than for bacteria, particularly in the gray pit (GP), which shows a predominance of very few species. Among the pit samples, the water sample (WP) showed the highest Shannon indices for fungi and bacteria, reaching values similar to those observed in soils. This highlights the role of water in transporting microorganisms from one ecosystem to another [38].

Simpson’s index varies between 0 and 1. A value close to 1 indicates low diversity, meaning that one or a few species dominate the community. In contrast, a value close to 0 indicates high diversity, with many species in relatively similar proportions. The microbial communities of the pits had significantly higher values than those of the soils, especially HR for fungi and GP for bacteria, which were strongly dominated by 1 or 2 genera. On the other hand, WP’s high values of the inverse Simpson index suggest a higher fungal and bacterial diversity, as well as increased fungal diversity in topsoils. This result indicates that WP has achieved a more even distribution of species.

In terms of OTU diversity, soils were characterized by a more significant number of phyla, classes, and genera than pits. Technosoils had more bacterial genera than “natural” soils but had fewer fungi. In general, a higher richness of soil microbiota is associated with a higher functional potential that translates into ecosystemic stability and adaptive capacity in the face of environmental perturbations [39]. However, some studies point out that a higher microbial richness does not necessarily reflect soil or ecosystem health [40]. This is especially important when amendments are added that introduce new microorganisms and potentially stimulate specific microbial processes, which may not be essential for soil recovery. It is, therefore, necessary to evaluate the roles played by members of the soil microbial communities before making decisions for soil restoration. These analyses could be assessed by metagenomic and transcriptomic studies that allow for measuring active biological processes [41].

UpSet Plot analyses at the class level revealed that more than half of the microbial classes were common to all soil samples (54.4% and 57.1% for bacteria and fungi, respectively), 54.8% for fungi in pits, but only 20% for bacteria in pits. Part of these communities could be considered a core microbiota, a set of microbial taxa widely distributed in different locations of a given habitat type. In the case of soils, the high degree of similarity is not so surprising because the topsoils come from pristine soils of the same area and enter the composition of the prepared soils (technosoils).

Interestingly, when comparing the microbiota present in pits and soils, it is observed that 49.2% of the classes are specific to soils, 45.8% are shared between pits and soils, and only 5.0% of the bacterial classes in pits are exclusive to pits (S1 dataset). At the genus level, this trend is maintained as 6.3% of the genera are pit-specific, 31.8% are found in both pits and soils, and 61.9% are exclusive to soils. A contrasting pattern emerged at the fungal class level, with 88.6% of the microbiota shared between soils and pits, while the remaining 11.4% was exclusively found in soils (S2 dataset). At the genus level, 4.4% were found only in pits, 23.9% in soils, and 71.7% were shared. These observations complement the results of the diversity indices and reinforce the idea that 1) at the bacterial level, soils exhibit greater diversity and exclusivity of classes and genera compared to pits. 2) For fungi, the same pattern is observed with the peculiarity that most species are shared between soils and pits.

On the other hand, it could be argued that the non-core microbiota originates from specific local events such as vegetation, physicochemical changes due to piling, or the introduction of amendments. The low proportion of shared bacterial microbiota in the pits could be related to specific crystal minerals/minerals that strongly influence the composition of these communities at the centimeter scale, with some microbes being restricted to one mineralogy [42, 43]. On the contrary, several authors reported that fungal communities are less sensitive to ecological variations and more stable over time despite having lower taxonomic diversity [44, 45], as described in this work.

Taxonomic analyses revealed dominant classes such as Acidobacteria and Verrucomicrobiae, as well as three subdivisions belonging to the group of Proteobacteria in soils. These results are consistent with previous studies highlighting the adaptability of these classes to soils impacted by mining [46, 47]. Proteobacteria mainly predominate in these mining environments [48]. In addition, Bacteroidetes, Ktedonobacteria, Actinomycetia, and Chitinophagia show notable variations in their distribution. While three classes harbor the core microbiota in pits, Gammaproteobacteria, Betaproteobacteria, and Alphaproteobacteria were highly predominant in most samples.

The community that characterizes these extreme environments is represented by nitrifying Chemolithoautotrophic bacteria such as the genus *Nitrosovibrio* or producers of volatile sulfur compounds such as *Holophaga* [49, 50]. Contrary to expectations, pristine soils present fewer genera, however, they are microorganisms that contribute significantly to ecosystems, such as the genera *Acidobacterium* or *Nitrosovibrio*, which participate in soil stability [51, 52]. On the other hand, among the technosoils, a considerable proportion of the genera *Bacteroides* and *Acidovorax* were observed. Bacteroides are part of the gastrointestinal microbiota of mammals and vegetated soils, and *Acidovorax*, whose name refers to the acidic degradation, is related to the reduction of nitrates [49,53,54]. The microbiota of the revegetated technosoil in San Pedro (TCP) showed the greatest diversity of interesting genera, being predominant *Thiobacillus*, which uses sulfur as an energy source; *Anaeromyxobacter*, which can fix nitrogen, *Geobacter*, with properties that make them useful in bioremediation, being the first organisms found with the capacity to oxidize organic compounds and metals; and *Geothrix*, which can reduce metals [55–58]. It is inferred that the greater diversity of genera found in technosoils is due to the amendments that introduce their microbiota added to the microorganisms added with the revegetation process in the case of TCP [59].

Among the bacterial microbiota of the pits, *Pseudomonas* predominates in the orange pit (POD, POW) and HR samples. *Pseudomonas* are ubiquitous species that exhibit high metabolic plasticity and can colonize a wide range of niches, even the most hostile [60]. Their ability to resist heavy metals and other pollutants means they are frequently found in contaminated ecosystems or employed as bioremediation species [61]. On the other hand, the dominant bacterium at 95% in the gray pits was *Burkholderia*, a saprophytic bacterium that also intervenes in the degradation of organic matter, with a high degradative capacity of organic contaminants [62]. Although they can be opportunistic pathogens for humans, animals, and plants, some strains seem resistant to heavy metals [63]. *Serratia* is ubiquitous in the environment, colonizing water, soil, plants, insects, animals, and food, particularly those rich in C [64]. Some strains found in mine tailings have been shown to utilize heavy metals to promote nitrogen fixation [65]. Among the most critical bacterial genera previously described, *Anaeromyxobacter*, *Geobacter*, *Bacteroides*, and *Holophaga* are the only ones identified exclusively in soils (S1 Dataset). Of the 28 bacteria specific to pits, only *Thiomonas* is a genus directly known for its ability to oxidize iron and sulfur and contribute to acid generation in the mining environment [66].

The few studies that have examined the fungal community structure of mining-impacted environments have shown that there are certain core members of the microbiota, such as Agaricomycetes, Leotiomycetes, and Pezizomycetes, that were critical in maintaining a stable ecological niche in both the root and rhizosphere [67, 68]. Furthermore, they point out that Leotiomycetes were the dominant class during the initial remediation period, but Pezizomycetes gradually became dominant as the remediation process progressed. In another research conducted in mine-contaminated soils, phylogenetic taxonomy showed that the most abundant fungi included Agaricomycetes, Nectriaceae, Eurotiomycetes, Mortierellaceae, Incertae sedis, Trichocomaceae, Sordariomycetes, and Fusarium [69].

The fungal community in the pristine soil was dominated by saprophytic genera such as *Archaeorhizomyces*, *Mortierella*, and *Sphaerosporella*, genera commonly found in soils. They are known for their interaction with plant roots and their role in decomposing complex organic substances, particularly plant residues. They are important in nutrient cycling and soil health. In particular, *Sphaerosporella* is found among mosses and bare soil [70–72].

In the pits, *Alatospora*, *Volutella*, and *Guehomyces* are found in the POD sample, although their presence is deficient in the other samples. The prevalence of *Cryptococcus* was detected in the GP sample. In the HR sample, the genus *Microbotryozyma* predominates. All these genera are saprophytes typically found in soil and decaying organic matter [73, 74]. In the case of fungi, all the major genera found in this study were present in both soils and pits, so they can be considered part of the core microbiota of the open pit environment.

The analysis of the less abundant genera grouped as “others” showed significant differences between samples only at the level of bacterial communities. The freshly prepared technosoil (TCZ), the revegetated technosoil (TCP), and the WP water sample showed the most dissimilar few dominant microbiota compared to the other samples. The WP sample was statistically the most dissimilar of all. These differences are speculated to be inherent to the ability of water to transport microorganisms from the ecosystems it passes through (soil, roots, pits) [75]. In addition, several studies have shown increased microbial diversity in soils receiving organic amendments [76], precisely like in the case of TCP. Moreover, the WP was the sample that regularly showed the highest microbial richness and diversity indices among the pits, with values close to or higher than those presented in the soils.

The biplot and Pearson analysis in the soils revealed two important lessons; 1) adding amendments to the topsoils strongly impacts the bacterial and fungal communities. 2) within the unamended soils, it is observed that the pristine soil is separated from the topsoils, and therefore, it is deduced that these soils have lost many characteristics of their original state. This should be contrasted with the profound physicochemical changes these soils undergo when displaced and piled, with the death and degradation of buried macro-organisms and with the apparition of oxygen, light and water gradients.

In this sense, the analysis of bacterial and fungal communities’ recovery in revegetated technosoils highlights contrasting dynamics and challenges in soil restoration. In bacterial communities, similarity to pristine soil (PSP) increased significantly, from 39.6% in freshly prepared technosoil (TCZ) to 59.8% after eight months of revegetation (TCP). This progress reflects the effectiveness of early restoration strategies, particularly for key classes such as Acidobacteria, Deltaproteobacteria, and Betaproteobacteria, which even exceeded PSP levels, suggesting their active role in stabilization and nutrient cycling. However, the persistence of “excessive prevalence” classes like Cytophagia and Chitinophagia, alongside the slow recovery of others such as Verrucomicrobia and Ktedonobacteria, indicates that certain bacterial taxa require longer recovery times or targeted interventions to restore their ecological functions. The significant proliferation of *Anaeromyxobacter* highlights its potential role as a bioindicator and facilitator in early soil recovery phases [77].

In contrast, fungal communities displayed slower recovery, with marginal increases in similarity to PSP. The proliferation of Sordariomycetes, which exceeded PSP levels, and the limited recovery of key classes such as Archaeorhizomycetes and Mortierellomycetes, indicate a more complex and prolonged recovery trajectory for fungi. At the genus level, the decrease in similarity, driven primarily by the decline of *Mortierella*, suggests that critical fungal taxa have yet to dominate restored soils. This slow progress may be linked to their greater sensitivity to environmental disturbances or competitive dynamics with opportunistic taxa such as Sordariomycetes [78]. Together, these findings emphasize the need for differentiated restoration approaches to promote the recovery of both bacterial and fungal communities, ultimately achieving ecological functionality comparable to pristine soils. To restore these soils effectively, pristine soils should be considered a reference and could serve as a source of inoculation of essential microorganisms for the restoration process, which have been lost or are scarce in the areas to be rehabilitated [79,80].

Our results show that specific soil physicochemical parameters, in particular pH, total nitrogen (TN) and, to a lesser extent, carbon (CO), are important drivers of microbial community composition. Adjustment of these parameters through targeted soil management could promote the development of specific microbial classes and genera. These interventions could be implemented during TCZ production or at different stages of technosoil maturation. For example, reducing pH while adding nitrogen and carbon to freshly prepared technosoil could promote key microbial taxa such as Verrucomicrobiae and Acidobacteria, which are essential for nutrient cycling [46,47], and fungal genera such as *Archaeorhizomyces*, which are critical for organic matter decomposition [70]. Nevertheless, the stabilization of certain microbial taxa, exemplified by Agaricomycetes, suggests that extended time rather than intervention may be sufficient to achieve PSP-like conditions. However, the dominance of certain fungal classes and genera observed exclusively in PSP highlights the need to include additional environmental parameters, such as heavy metals, sulfate presence, soil texture and moisture, to refine our understanding of microbial dynamics in technosoil ecosystems.

Balancing the microbial community in technosoils must also ensure the establishment of optimal conditions for rapid vegetation recovery. Vegetative cover plays a protective role by mitigating soil erosion, reducing leachate generation, and minimizing acid water infiltration [81]. Thus, future strategies should integrate microbial and vegetative restoration efforts to accelerate ecosystem recovery while maintaining soil stability.

For pits, the ecological similarities analysis reflected how fungi community structure is directly influenced by 1) moisture conditions (wet pit POW – water collected WP, different from dry pit POD), 2) different rock compositions or sulfur content (GP and HR), and 3) disturbance caused by traffic (HR). No clear pattern of similarity in the prokaryotic communities could be observed or matched with the above characteristics. We can speculate that the variation of similarities between clusters of bacterial and fungal communities is due to the “extreme” environment represented by these pits, exposed to dust, weathering, lack of nutrients, solar radiation, and extreme pH, among others [34]. It is also very likely that variations strongly influence the variations in bacterial community structures observed in our study in the mineral composition of the pit [41,42,82]. The diversity and richness of fungi compared to bacteria can be attributed to several factors 1) fungi are generally more resistant to desiccation and extreme temperature and pH fluctuations than many bacteria. 2) Fungi can form spores that enable them to survive in adverse conditions for extended periods. 3) They possess a greater capacity for obtaining complex nutrients through decomposition or their ability to form extensive mycelia, which allows them to explore and colonize vast areas, thereby gaining an advantage in environments where nutrients are scarce [83].

Due to their extension and verticality, remediation options for pits are more limited than for soils. Combining the inoculation of pit ubiquitous microorganisms with amendments would promote the formation of a stable, protective biofilm to prevent the formation of acidic water and heavy metal drainage. The results of this study indicate that an increase in both fungal and bacterial diversity would be necessary to achieve an ecosystem multifunctionality comparable to that observed in soils.

## Conclusion

This manuscript presents the findings of a comprehensive study investigating the microbial communities in diverse soil types and pit ecosystems associated with open-pit gold mine environments in Peru. A metabarcoding approach was utilized to analyze the bacterial and fungal compositions across five distinct soil categories and different pit samples. The findings reveal significant differences in microbial richness, diversity, and community composition between pristine soils, topsoils and various technosoils, as well as a stark contrast between soil samples and pit environments. In particular, the findings demonstrate that pristine and stockpiled topsoils exhibit higher microbial richness, followed by technosoils. Pits show the most reduced diversity and are characterized as extreme ecosystems that limit microbial growth. The study demonstrates that bacterial diversity is superior to fungal diversity in soil samples, whereas this is not the case in pit samples. The results also indicate that the addition of amendments affects soil microbial ecosystems, and that these ecosystems remain less complex than those in pristine soils, potentially due to their new physicochemical properties. However, the vegetablization process tends to rebalance these communities with those of pristine soils over time. Runoff water was demonstrated to contribute to this rebalancing by transporting microorganisms from one ecosystem to another. The results also highlight the critical role of soil physicochemical parameters, such as pH, nitrogen and carbon content, in shaping microbial communities. Effective management of these parameters could enhance microbial recovery by promoting conditions similar to those found in pristine soils. The research presented provides valuable insights into the dynamics of soil and pit microbial communities in the context of mining activities. It provides a scientific basis for future restoration strategies aimed at improving microbial diversity and ecosystem resilience in altered landscapes.

## Supporting information

S1 Table Sampling points information. CMC; Minera Coimolache S.A., MLZ; Minera La Zanja. C.O: Organic Carbon; T.N: Total Nitrogen; T.C: Total Carbon; T.S: Total Sulfur. (XLSX)

S2 Table Diversity indices and sequence processing summary of 16S rRNA metagenomic analysis in soil and pit samples at class and genus levels. PSP: Pristine soil, TSD; topsoil from Deposit 1, TSO; topsoil from the DMO deposit, TSC; topsoil from the Coimolache deposit, TCZ; recently amended topsoil (technosoil), TCP; eight-month-old amended technosoil. POD; Dry orange pit, POW; wet orange pit, GP; dry gray pit, HR; hold road, WP; water collected from the pit. (XLSX)

S3 Table Diversity indices and sequence processing summary of ITS metagenomic analysis in soil and pit samples at class and genus levels. PSP: Pristine soil, TSD; topsoil from Deposit 1, TSO; topsoil from the DMO deposit, TSC; topsoil from the Coimolache deposit, TCZ; recently amended topsoil (technosoil), TCP; eight-month-old amended technosoil. POD; Dry orange pit, POW; wet orange pit, GP; dry gray pit, HR; hold road, WP; water collected from the pit.
(XLSX)

S4 Table Number of bacterial and fungal OTUs observed in soil and pit samples. PSP: Pristine soil, TSD; topsoil from Deposit 1, TSO; topsoil from the DMO deposit, TSC; topsoil from the Coimolache deposit, TCZ; recently amended topsoil (technosoil), TCP; eight-month-old amended technosoil. POD; Dry orange pit, POW; wet orange pit, GP; dry gray pit, HR; hold road, WP; water collected from the pit. (XLSX)

S5 Table Comparative prevalence and RPKM analysis of bacterial classes in soil samples, highlighting classes with prevalence > 1% (A) and all detected classes (B). Green cells indicate prevalences higher than those in PSP while numbers in red indicate negative values,.PSP: Pristine soil, TCZ; recently amended topsoil (technosoil), TCP; eight-month-old amended technosoil. (XLSX)

S6 Table Comparative prevalence and RPKM analysis of bacterial genera in soil samples, highlighting genera with prevalence > 1% (A) and all detected genera (B). Green cells indicate prevalences higher than those in PSP while numbers in red indicate negative values,.PSP: Pristine soil, TCZ; recently amended topsoil (technosoil), TCP; eight-month-old amended technosoil. (XLSX)

S7 Table Comparative prevalence and RPKM analysis of fungii classes in soil samples, highlighting classes with prevalence > 1% (A) and all detected classes (B). Green cells indicate prevalences higher than those in PSP while numbers in red indicate negative values,.PSP: Pristine soil, TCZ; recently amended topsoil (technosoil), TCP; eight-month-old amended technosoil. (XLSX)

S8 Table Comparative prevalence and RPKM analysis of fungii genera in soil samples, highlighting genera with prevalence > 1% (A) and all detected genera (B). Green cells indicate prevalences higher than those in PSP while numbers in red indicate negative values,.PSP: Pristine soil, TCZ; recently amended topsoil (technosoil), TCP; eight-month-old amended technosoil. (XLSX)

S1 Fig Canonical Correspondence Analysis (CCA) showing the distribution of major bacterial classes (>2% prevalence) in relation to key soil variables, including pH, organic carbon content (C.O), and total nitrogen content (T.N.). *PSP*: Pristine soil, *TSD*; topsoil from Deposit 1, *TSO*; topsoil from the DMO deposit, *TSC*; topsoil from the Coimolache deposit, *TCZ*; recently amended topsoil (technosoil), *TCP*; eight-month-old amended technosoil. (TIF)

S2 Fig Canonical Correspondence Analysis (CCA) showing the distribution of major bacterial genera (>2% prevalence) in relation to key soil variables, including pH, organic carbon content (C.O), and total nitrogen content (T.N.). *PSP*: Pristine soil, *TSD*; topsoil from Deposit 1, *TSO*; topsoil from the DMO deposit, *TSC*; topsoil from the Coimolache deposit, *TCZ*; recently amended topsoil (technosoil), *TCP*; eight-month-old amended technosoil. (TIF)

S3 Fig Canonical Correspondence Analysis (CCA) showing the distribution of major fungal classes in relation to key soil variables, including pH, organic carbon content (C.O), and total nitrogen content (T.N.). *PSP*: Pristine soil, *TSD*; topsoil from Deposit 1, *TSO*; topsoil from the DMO deposit, *TSC*; topsoil from the Coimolache deposit, *TCZ*; recently amended topsoil (technosoil), *TCP*; eight-month-old amended technosoil. (TIF)

S4 Fig Canonical Correspondence Analysis (CCA) showing the distribution of major fungal genera (>2% prevalence) in relation to key soil variables, including pH, organic carbon content (C.O), and total nitrogen content (T.N.). *PSP*: Pristine soil, *TSD*; topsoil from Deposit 1, *TSO*; topsoil from the DMO deposit, *TSC*; topsoil from the Coimolache deposit, *TCZ*; recently amended topsoil (technosoil), *TCP*; eight-month-old amended technosoil. (TIF)

S1 Dataset Distribution of common and exclusive bacterial classes and genera in pit and soil samples.
Genera cited in the text are highlighted in yellow.
(XLSX)

S2 dataset Distribution of common and exclusive bacterial classes and genera in pit and soil samples.
Genera cited in the text are highlighted in yellow.
(XLSX)
