# Supplementary figures and images for: Assessing microbial diversity in open-pit mining: Metabarcoding analysis of soil and pit microbiota across operational and restoration stages
Source: PLoS One. 2025 Apr 7;20(4):e0320923. doi: 10.1371/journal.pone.0320923 (PMC11975129; doi:10.1371/journal.pone.0320923)

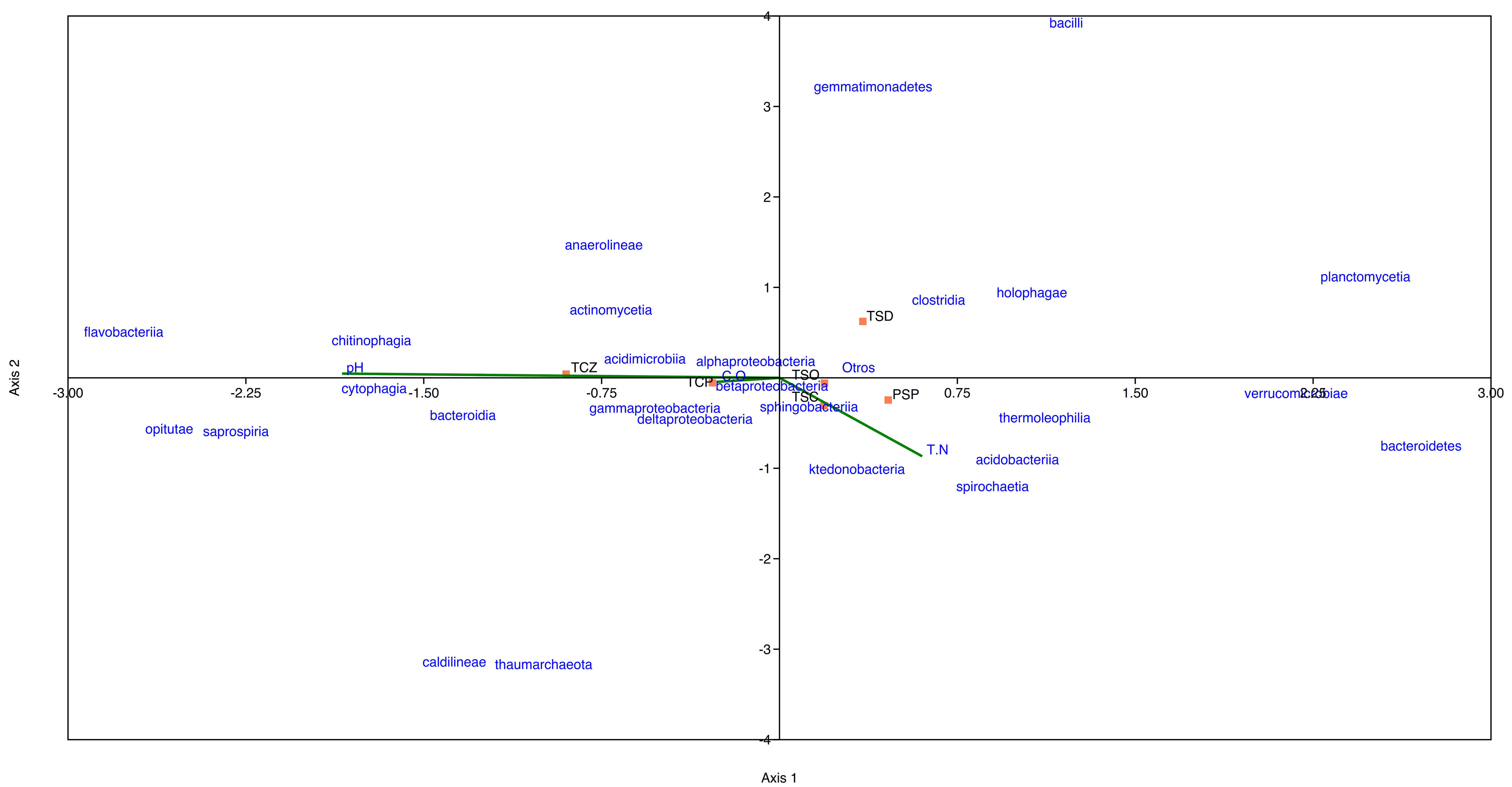

Supplement: S1 Fig — (TIF) [file pone.0320923.s009.tif]

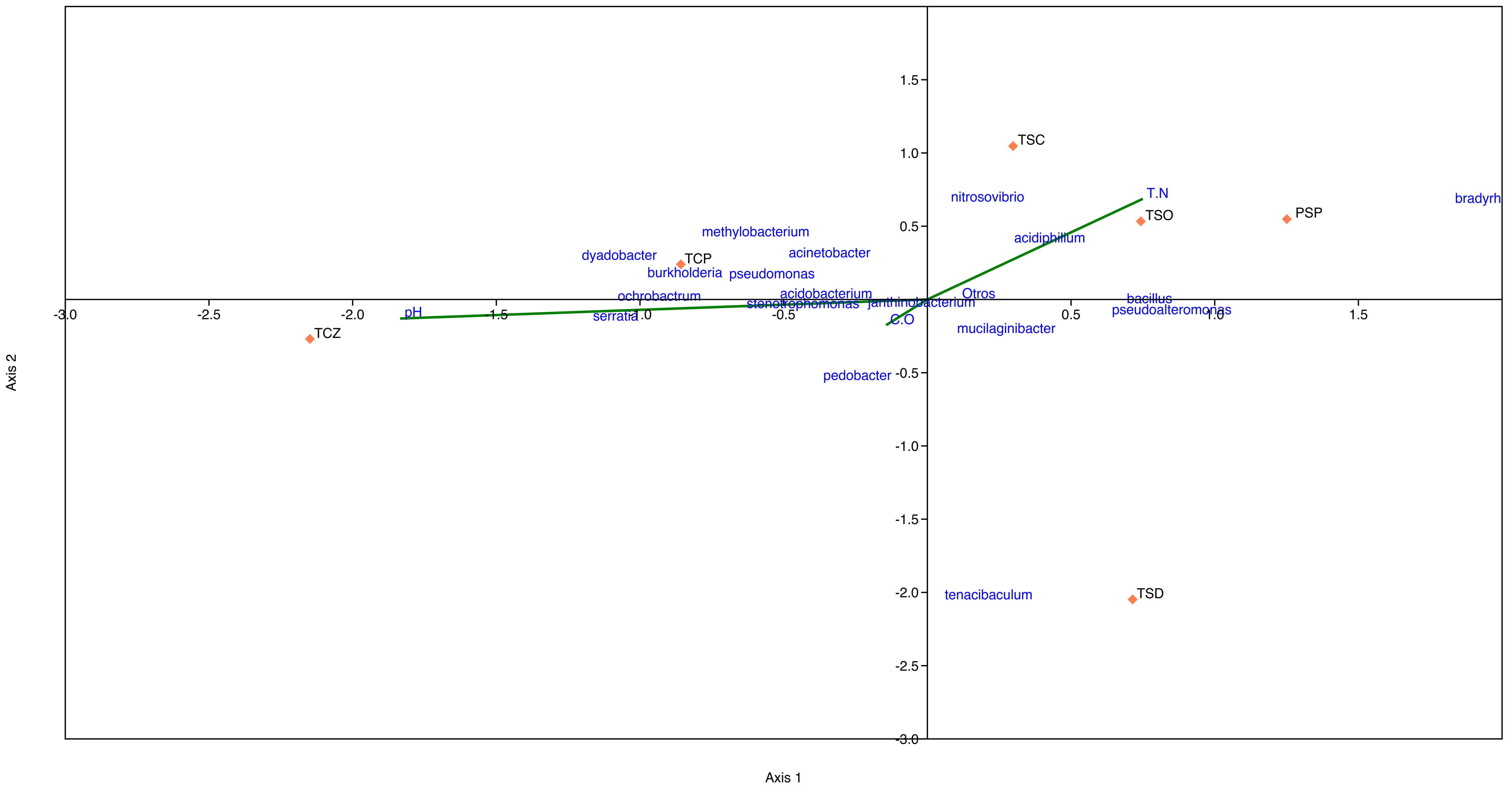

Supplement: S2 Fig — (TIF) [file pone.0320923.s010.tif]

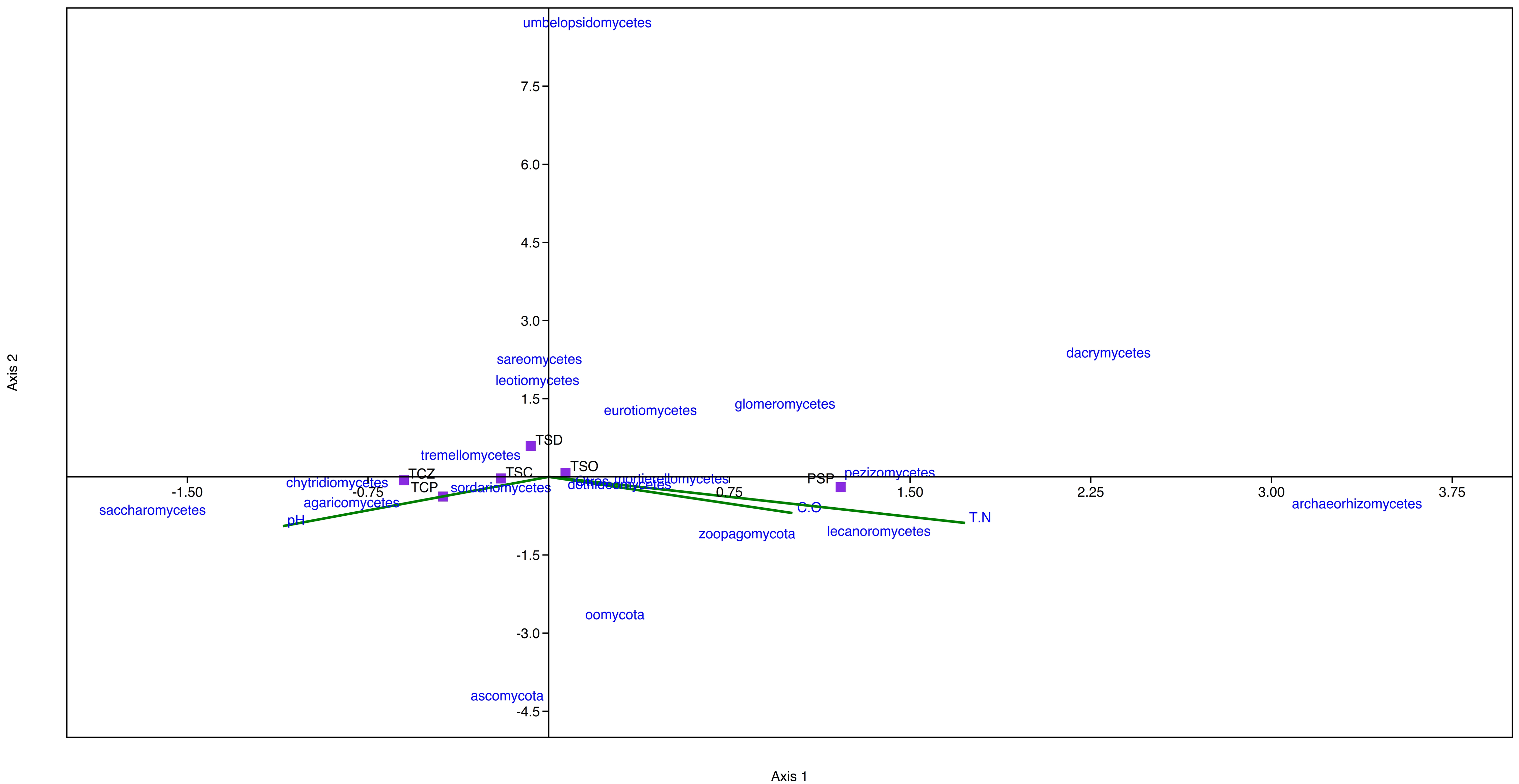

Supplement: S3 Fig — (TIF) [file pone.0320923.s011.tif]

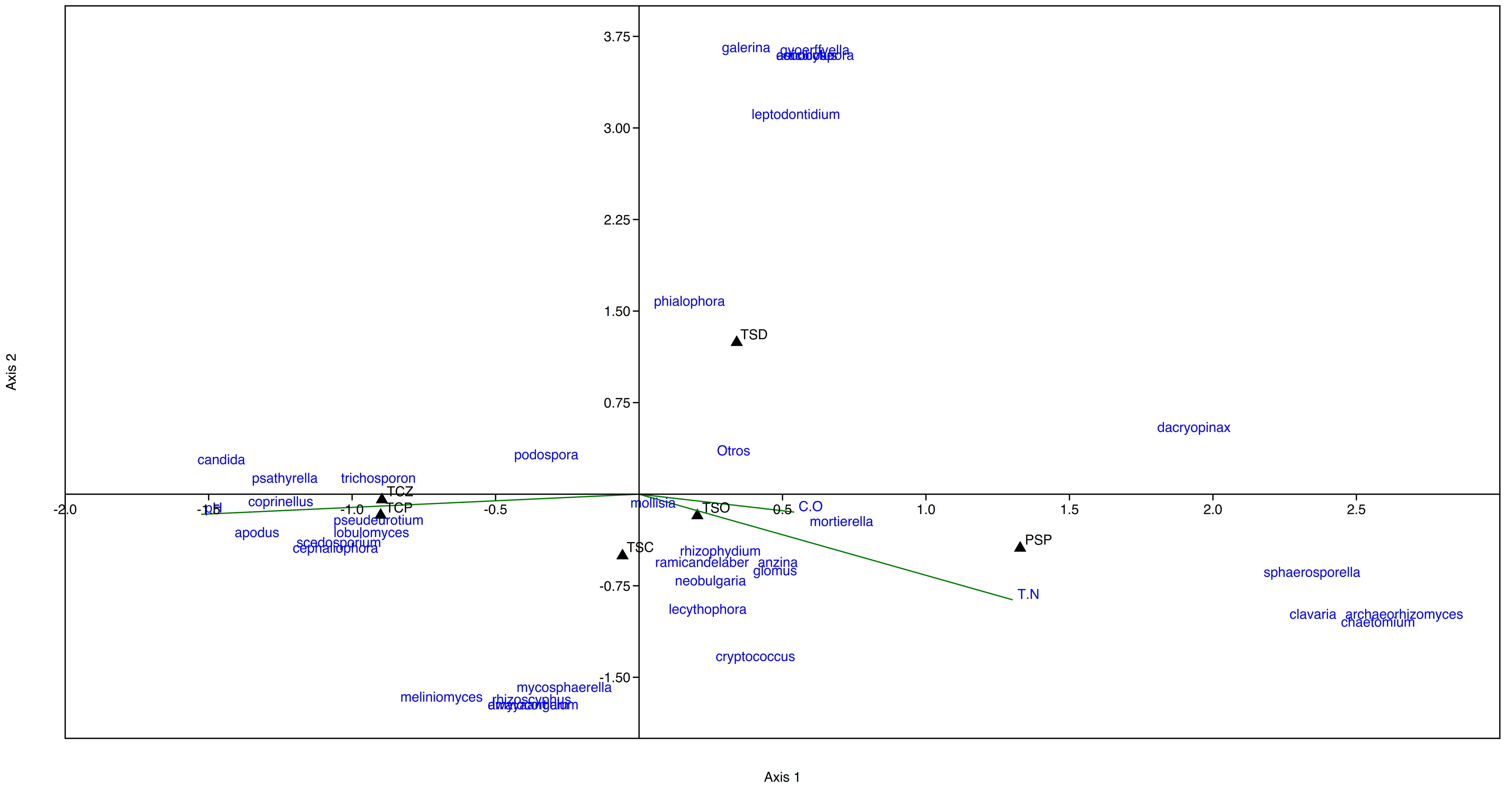

Supplement: S4 Fig — (TIF) [file pone.0320923.s012.tif]
